# Paroxysmal Nocturnal Hemoglobinuria: Unraveling Its Molecular Pathogenesis and Advancing Targeted Therapeutic Strategies

**DOI:** 10.3390/diseases13090298

**Published:** 2025-09-09

**Authors:** Elisavet Apostolidou, Vasileios Georgoulis, Dimitrios Leonardos, Eleni Kapsali, Eleftheria Hatzimichael

**Affiliations:** Department of Haematology, Faculty of Medicine, School of Health Sciences, University of Ioannina, St. Niarchou Av., 45 500 Ioannina, Greece; e.apostolidou@uoi.gr (E.A.); v.georgoulis@uoi.gr (V.G.); d.leonardos@uoi.gr (D.L.); ekapsali@uoi.gr (E.K.)

**Keywords:** paroxysmal nocturnal hemoglobinuria, complement inhibitors, intravascular hemolysis, extravascular hemolysis, breakthrough hemolysis

## Abstract

Paroxysmal nocturnal hemoglobinuria (PNH) is a rare, acquired clonal hematologic disorder caused by somatic mutations in the *PIGA* gene of hematopoietic stem cells, leading to the absence of GPI-anchored proteins, including the complement regulators CD55 and CD59. This deficiency results in uncontrolled complement activation, causing intravascular and extravascular hemolysis, thrombosis, and bone marrow failure. Historically associated with substantial morbidity, PNH management has been transformed by the advent of complement inhibitors. Eculizumab, the first approved C5 inhibitor, significantly reduced thrombotic risk and improved survival but did not eliminate anemia due to extravascular hemolysis. Newer agents now target proximal complement components, offering broader control and improved convenience. This review summarizes the pathophysiology of PNH, evaluates established and emerging complement inhibitors, and discusses ongoing therapeutic challenges and future directions.

## 1. Introduction

Paroxysmal nocturnal hemoglobinuria (PNH) represents a paradigm of acquired clonal hematopoietic disorders with immune-mediated pathogenesis through overactivation of the complement cascade [1]. The estimated annual incidence of PNH ranges from 3.5 to 5.7 cases per million, with a prevalence of 13 to 38 per million and a median age of onset between 35 and 40 years [2].

At the core of PNH pathogenesis lies a somatic mutation in the phosphatidylinositol glycan class A (*PIGA*) gene in hematopoietic stem cells, disrupting GPI-anchor biosynthesis and leading to the absence of complement regulatory proteins such as CD55 and CD59 on the cell surface [1,2]. This defect allows unregulated complement activation, with red blood cells (RBCs) particularly vulnerable to destruction by the membrane attack complex (MAC) leading to intravascular hemolysis (IVH), whereas white blood cells (WBCs) and platelets contribute to thrombophilic state and infectious risk [3]. PNH is described as a “chameleon-like” disease due to its wide spectrum of clinical manifestations, variable severity, and its ability to mimic other hematologic disorders, often complicating the diagnostic process. The clinical presentation includes the classic triad of chronic intravascular hemolytic anemia, thrombosis and bone marrow failure. Thromboembolic events are often the first manifestation of the disease and represent the leading cause of morbidity and mortality. The pathogenesis is multifactorial, involving complement-mediated platelet activation, hemolysis with nitric oxide depletion, endothelial dysfunction, neutrophil activation and release of procoagulant microparticles, and typically affects unusual venous sites such as intra-abdominal or hepatic veins [4]. Beyond these hallmark features, hemoglobinuria represents a distinctive clinical sign, albeit variable, manifesting as dark-colored urine most noticeable in the morning. Smooth muscle dystonia, resulting from impaired nitric oxide bioavailability due to free hemoglobin released during IVH, contributes to abdominal pain, esophageal spasm, and erectile dysfunction. Renal involvement ranges from acute injury to chronic impairment, as sustained hemolysis causes hemosiderin deposition and impairs proximal tubular function. Additional complications include pulmonary hypertension, vascular dysfunction with manifestations such as dysphagia and dyspnea, and an increased risk of maternal–fetal morbidity during pregnancy, mainly due to thrombosis, miscarriage, and preterm delivery. Among the diverse manifestations of PNH, fatigue is the most prevalent and serves as a key determinant of patients’ quality of life, reflecting both the burden of chronic hemolysis and the psychological impact of a lifelong disease [5,6,7]. The diagnosis of PNH depends on confirmation of the deficiency of at least two distinct GPI proteins, mainly CD55 and CD59, within two separate cell lineages (granulocytes, erythrocytes and monocytes) based on flow cytometry in peripheral blood sample [1,3].

Prior to the development of complement inhibitors, PNH carried a high risk of morbidity and mortality, with a 5-year survival rate of only 35% due to complications such as hemolysis, thrombosis, and marrow failure [8]. Until the end of the last century, the treatment options were restricted to supportive care, while allogenic hematopoietic stem cell transplantation (HSCT) was the sole curative treatment mostly for younger and fit patients, albeit with high associated risks [8,9]. The introduction of complement inhibitors has revolutionized the field of treatment in PNH, significantly improving patient outcomes and quality of life. Starting with the approval of eculizumab in 2007, the complement inhibitors succeed in controlling the PNH effect without offering a complete cure, while challenges remain, including residual extravascular hemolysis (EVH) control, thrombotic risk, and accessibility issues due to cost and logistics [10,11]. Regarding these unmet needs, multiple therapeutic options have emerged, targeting different pathways in the complement cascade.

## 2. Materials and Methods

This narrative review was conducted through a comprehensive literature search of PubMed, Scopus, and ClinicalTrials.gov databases covering publications from January 2005 through May 2025. The search strategy employed combinations of key terms such as “Paroxysmal Nocturnal Hemoglobinuria,” “complement inhibitors,” “eculizumab,” “ravulizumab,” “pegcetacoplan,” “proximal complement blockade,” and “clinical trials.” Inclusion criteria encompassed peer-reviewed original research articles, pivotal Phase II/III clinical trial reports, regulatory submissions, and expert consensus guidelines in English. Studies were selected based on clinical relevance, methodological quality, and contribution to evolving therapeutic paradigms. References were cross-checked manually to ensure completeness and coverage of all major complement-targeting therapies, both approved and investigational.

## 3. Disease Overview

The phenotype of PNH arises from acquired somatic mutations in the *PIGA* gene, located on the X chromosome. This gene encodes a glycosyltransferase essential for the initial step in the biosynthesis of glycosylphosphatidylinositol (GPI) anchors. A deficiency in GPI anchors results in partial or complete absence of all GPI-anchored proteins (GPI-APs), including key complement regulatory proteins such as CD55 (decay-accelerating factor) and CD59 (membrane inhibitor of reactive lysis) [1,12].

CD59 inhibits the formation of the MAC, while CD55 accelerates the decay of C3 convertases, both acting as protective mechanisms against complement-mediated damage [12,13]. In PNH, RBCs become highly vulnerable to MAC-mediated intravascular lysis. In contrast, WBCs and platelets retain other complement regulatory proteins such as CD46, which offer partial protection, although their role in promoting thrombosis and immune dysregulation remains significant [14].

The complement system, a central component of innate immunity, plays a crucial role in the pathogenesis of PNH. Complement activation proceeds via three distinct pathways—classical (CP), lectin (LP), and alternative (AP), each triggered by different stimuli and following a multistep cascade [15].

The CP is initiated by the binding of C1q to the Fc region of immunoglobulins, culminating in the formation of the C3 convertase (C4b2a). The LP is activated through recognition of specific carbohydrate patterns on microbial surfaces by mannan-binding lectin (MBL) or ficolins, which similarly lead to the formation of C3 convertase [16,17]. The AP is distinct in that it is constitutively active at a low level through the spontaneous hydrolysis of C3, a process termed “C3 tick-over.” This pathway includes a positive feedback loop that amplifies C3b generation and continuously surveys for invading pathogens. Due to its central role in C3-mediated EVH, the AP was the first therapeutic target in modern PNH treatment strategies [16].

All three pathways converge at C3, leading to the generation of C3 and C5 convertases. These complexes mediate the cleavage of C5 into C5a (an anaphylatoxin) and C5b, the latter initiating assembly of the MAC, which culminates in cell lysis [18].

## 4. Diagnosis

The diagnosis of PNH is based on a combination of clinical assessment and laboratory investigations. Traditional markers of hemolysis, such as lactate dehydrogenase (LDH), reticulocyte counts, plasma free hemoglobin, and haptoglobin, provide important information on intravascular red cell destruction but lack diagnostic accuracy. Flow cytometric immunophenotyping remains the gold standard, enabling the detection of GPI-deficient cells across multiple lineages, namely granulocytes, monocytes, and RBCs, with high sensitivity and specificity. The use of fluorescent aerolysin (FLAER)–based assays combined with next-generation multicolor flow cytometry (MFC) has significantly improved diagnostic accuracy for PNH. These advances enable detection of even very small PNH clones and enhance both initial diagnosis and longitudinal monitoring.

In addition to classical hemolysis markers, researchers are investigating emerging biomarkers that may better reflect disease activity, predict therapeutic response, and monitor treatment efficacy. These include C3 fragment deposition on erythrocytes, free C5 levels, soluble terminal complement complex (sC5b-9), and plasma free hemoglobin levels. However, standardization of these biomarkers is necessary before they can be fully integrated into routine clinical practice [19,20].

**Figure 1:**
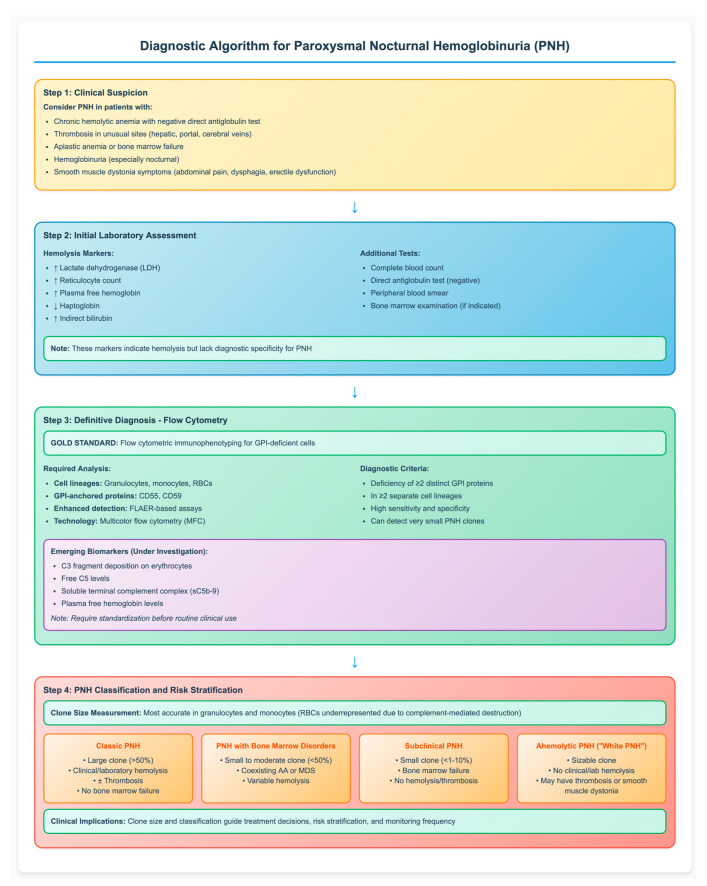
Diagnostic Algorithm for Paroxysmal Nocturnal Hemoglobinuria (PNH). The flowchart illustrates the step-by-step approach to PNH diagnosis, from clinical suspicion through definitive flow cytometric confirmation to disease classification. Flow cytometry remains the gold standard, requiring demonstration of GPI-deficient cells in multiple lineages. Clone size determines clinical phenotype and guides treatment decisions. AA: aplastic anemia; MDS: myelodysplastic syndromes; FLAER: fluorescent aerolysin; GPI: glycosylphosphatidylinositol; RBC: red blood cell, ↑: increased; ↓: decreased.

The detected PNH clone strongly influences clinical manifestations and disease course, depending largely on its size and association with bone marrow failure. The clone size is most accurately measured in granulocytes and monocytes, as RBCs are often underrepresented due to ongoing complement-mediated destruction. Based on clinical studies, large PNH clones are associated with hemolysis, symptom burden, and thrombotic risk, thus defining the subgroup of patients most likely to benefit from complement inhibitors. In contrast, smaller PNH clones are subclinical and usually occur in patients with cytopenia or bone marrow failure, lacking clinical hemolysis [21]. According to the International PNH Interest Group, PNH is classified into three primary clinical subtypes based on clone size and associated features: (a) Classic PNH: characterized by a large clone size (>50%) with clinical or laboratory evidence of hemolysis and/or thrombosis in the absence of bone marrow failure; (b) PNH associated with bone marrow disorders: defined by a small to moderate clone size (<50%) coexisting with conditions such as aplastic anemia (AA) or myelodysplastic neoplasms (MDS) (c) Subclinical PNH: involves small clones (<1–10%) in patients with bone marrow failure, typically without hemolysis or thrombosis [21,22]. Recently, a fourth phenotype referred to as “ahemolytic PNH” or “white PNH” has been described. This variant is defined by the presence of a sizable PNH clone in the absence of clinical or laboratory evidence of hemolysis but may still be associated with symptoms such as thrombosis or smooth muscle dystonia [23]. Recognizing the association between clone size and clinical phenotype is essential for accurate risk stratification, appropriate timing of therapy initiation, and effective long-term disease monitoring. Figure 1 provides a comprehensive diagnostic algorithm that integrates clinical presentation, laboratory assessment, and flow cytometric analysis for the systematic evaluation of suspected PNH cases.

## 5. Evolvement in Complement Inhibition

The initiation of complement inhibitor therapy in PNH is primarily indicated in patients with clinically significant IVH and/or thromboembolic events, typically associated with a large PNH clone and high disease burden.

The therapeutic approach to PNH has become increasingly personalized, based on the predominant clinical phenotype, whether hemolysis, thrombosis, or bone marrow failure and informed by both approved and investigational agents. Patients are commonly stratified into three clinical categories:

### 5.1. Hemolysis-Predominant PNH

Patients with subclinical PNH, characterized by small clones and no clinical or biochemical evidence of hemolysis, do not require immediate treatment but should undergo periodic monitoring every 6-12 months. In contrast, patients with classic PNH, with active IVH, are eligible for complement inhibition, typically with terminal C5 inhibitors (C5i) such as eculizumab, ravulizumab, or crovalimab, or alternatively with pegcetacoplan, a proximal C3 inhibitor (C3i). In cases of inadequate response or breakthrough hemolysis, further options include: eculizumab biosimilars, iptacopan (a factor B inhibitor), danicopan (a factor D inhibitor, as an add-on to ravulizumab) or combination strategies (e.g., C5i + C3i) which are currently under investigation in clinical trials.

### 5.2. Thrombosis-Predominant PNH

Management requires a dual approach combining anticoagulation with complement inhibition, given the central role of complement-mediated platelet activation in thrombogenesis.

### 5.3. PNH with Bone Marrow Failure Syndromes

In patients with coexisting bone marrow failure (e.g., aplastic anemia or myelodysplastic syndromes), treatment decisions are guided by the underlying disease. Young patients with PNH and aplastic anemia (AA) may benefit from immunosuppressive therapy and/or allogeneic HSCT. In cases associated with MDS or AML, disease-specific therapy and consideration for HSCT are warranted.

**Figure 2:**
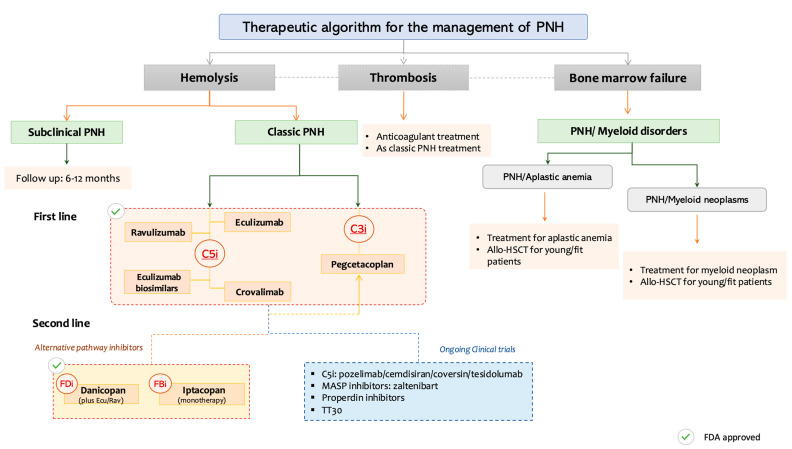
Personalized therapeutic approach to paroxysmal nocturnal hemoglobinuria (PNH) based on the dominant clinical phenotype. PNH: Paroxysmal nocturnal hemoglobinuria, C3i: C3 inhibitors, C5i: C5 inhibitors, FDi: Factor D inhibitors, FBi: Factor B inhibitors, Allo-HSCT: Allogeneic Hematopoeitic Stem Cell Transplantation, MASP: Mannan-binding lectin-associated serine protease.

Figure 2 provides a practical, phenotype-driven algorithm for PNH management, categorizing patients based on dominant clinical features: hemolysis, thrombosis, or coexisting bone marrow failure, and aligning these with targeted therapeutic options, including approved agents and those in advanced clinical development.

This decision framework categorizes patients based on predominant clinical manifestations: subclinical PNH, classic hemolytic PNH, thrombosis-predominant PNH, and PNH with bone marrow failure syndromes. Treatment strategies are stratified by phenotype and integrate both approved and investigational agents. C5 inhibitors (eculizumab, ravulizumab, crovalimab), C3 inhibitors (pegcetacoplan), and proximal AP blockers (factor B and factor D inhibitors) are mapped accordingly. BTH and treatment sequencing options (e.g., C5i + C3i combinations) are also noted. Clinical trial status and FDA approval are denoted by respective icons.

### 5.4. Terminal Complement Inhibitors

Until recently, C5 inhibitors (C5i) have represented the standard of care for the treatment of PNH. These monoclonal antibodies primarily target terminal complement activation, thereby preventing C5 cleavage into C5a and C5b and ultimately inhibiting the formation of the MAC and the intravascular lysis of RBCs [1,24]. However, persistent anemia remains a significant clinical challenge in a subset of patients treated with C5 inhibitors, primarily due to EVH, a phenomenon driven by C3 fragment deposition on RBCs and their subsequent removal by the mononuclear phagocyte system [25].

#### 5.4.1. Eculizumab

Eculizumab, the first terminal complement inhibitor approved for the treatment of PNH is a humanized monoclonal antibody that targets complement component C5, preventing its cleavage into C5a and C5b. This inhibition blocks the formation of the MAC (C5b-9), thereby reducing IVH [10]. The U.S. FDA has approved eculizumab for the treatment of PNH in adults, children and during pregnancy [10,11]. Its approval was based on the pivotal TRIUMPH trial, a Phase III double-blind, placebo-controlled study, which demonstrated that eculizumab significantly reduced IVH and transfusion requirements. The primary endpoints included stabilization of hemoglobin levels, while secondary outcomes showed reductions in thromboembolic events and improvements in quality of life, with an acceptable safety profile [26,27,28]. Further evidence was provided by the SHEPHERD trial, an open-label, multicentre Phase III study involving a broader population of PNH patients—which reaffirmed the efficacy and tolerability of eculizumab over a 52-week period, following the dosing schedule established in TRIUMPH [26,27,28].

Despite its efficacy, eculizumab treatment is associated with breakthrough hemolysis (BTH), a recurrence of IVH due to incomplete complement inhibition. BTH may result from subtherapeutic drug levels (pharmacokinetic BTH) or increased complement activation under stress, such as infection or surgery (pharmacodynamic BTH) [29,30,31]. Additionally, approximately 50% of patients treated with C5 inhibitors develop EVH, attributed to C3d deposition on RBCs, leading to persistent anemia and transfusion dependency [32]. Another important concern is the increased susceptibility to infections with encapsulated bacteria, particularly Neisseria meningitidis, Haemophilus influenzae, and Streptococcus pneumoniae. To mitigate this risk, patients should receive appropriate vaccinations at least two weeks prior to initiating C5 inhibitor therapy [33]. Finally, the requirement for biweekly intravenous administration presents significant logistical challenges and limits patient convenience. Moreover, the inability to address EVH and BTH underscores the need for newer therapeutic strategies or adjunctive agents [10].

#### 5.4.2. Ravulizumab

Ravulizumab is a long-acting, recombinant C5 inhibitor (C5i) derived from eculizumab. It was specifically engineered to overcome key limitations of its predecessor, including the short half-life, the requirement for frequent intravenous infusions, and suboptimal EVH control [28]. The enhanced pharmacokinetic and pharmacodynamic profile of ravulizumab is due to four targeted amino acid substitutions, which promote endosomal dissociation of the ravulizumab–C5 complex. This process facilitates C5 degradation in lysosomes. In parallel, ravulizumab’s increased affinity for the neonatal Fc receptor (FcRn) allows the antibody to be recycled back into circulation, thereby prolonging its half-life [28].

The efficacy and safety of ravulizumab were confirmed in two pivotal Phase III trials: Study 301 in treatment-naïve patients and Study 302 in patients previously treated with eculizumab. Both studies demonstrated that ravulizumab was non-inferior to eculizumab, with the additional benefit of extended 8-week dosing intervals [34,35]. Ravulizumab achieved complete terminal complement inhibition from the first infusion, maintaining consistently low serum free C5 levels throughout treatment. Clinical outcomes included sustained control of IVH, transfusion independence, thrombosis prevention, and a comparable safety profile to eculizumab, with fewer BTH events reported [36,37]. Adverse events were generally mild to moderate, although meningococcal infection remains a primary safety concern, necessitating appropriate vaccination [35,38]. A recent long-term analysis with follow-up of up to six years reaffirmed ravulizumab as a durable and well-tolerated treatment, with favorable survival outcomes and no new safety signals [37].

#### 5.4.3. Crovalimab

Crovalimab is a next-generation, subcutaneously administered C5i designed using recycling antibody technology with pH-dependent binding. Unlike eculizumab and ravulizumab, crovalimab targets a distinct epitope on the C5 molecule but retains the core mechanism of blocking terminal complement activation. Its molecular design allows for repeated binding to C5, enabling prolonged complement inhibition at lower doses and facilitating once-monthly subcutaneous administration, thus improving treatment convenience [31,39].

The efficacy and safety of crovalimab were evaluated in three large, multinational, randomized Phase III clinical trials—COMMODORE 1, COMMODORE 2, and COMMODORE 3—in both eculizumab-experienced and complement inhibitor-naïve PNH populations. COMMODORE 1 demonstrated that crovalimab was well tolerated and provided sustained terminal complement inhibition in patients transitioning from eculizumab [40]. COMMODORE 2 confirmed the non-inferiority of crovalimab compared to eculizumab in treatment-naïve patients while maintaining a favorable safety profile [41]. COMMODORE 3, a multicentre Phase III study in an Asian population, showed reduced transfusion dependency, adequate hemolysis control, and low rates of BTH, with only a single BTH event recorded [40]. Collectively, these results position crovalimab as a highly effective, less invasive alternative to traditional intravenous C5i, with the advantages of subcutaneous self-administration, less frequent dosing, and a favorable risk–benefit profile [31,42].

### 5.5. Proximal Complement Inhibitors

The need to address residual IVH and particularly C3-mediated EVH has driven the development of novel agents that target the proximal components of the complement cascade. Unlike terminal inhibitors (e.g., eculizumab and ravulizumab), these agents block upstream events, such as C3 activation, or inhibit key enzymatic components of the alternative pathway (AP), including factor B (FB) and factor D (FD) [3].

#### 5.5.1. Pegcetacoplan

Pegcetacoplan is a subcutaneous, pegylated, first-in-class C3 inhibitor developed to address the limitations of C5 inhibitors, particularly C3-mediated EVH. By blocking complement activation upstream of C5, pegcetacoplan offers broader inhibition of the complement cascade, thereby controlling both IVH and EVH [43].

The drug binds to C3 and C3b, preventing the formation and deposition of C3b on RBCs. It acts at multiple levels within the AP, including the tick-over mechanism and the assembly of C3 and C5 convertases, thereby suppressing both the amplification loop and terminal activation [44,45]. Pegcetacoplan is FDA-approved for use in treatment-naïve patients and in those previously treated with eculizumab or ravulizumab [46].

The pivotal PEGASUS trial—a Phase III, open-label, active-comparator study—demonstrated the superiority of pegcetacoplan over eculizumab in patients with hemoglobin levels <10.5 g/dL. The trial achieved its primary endpoint, showing a greater improvement in hemoglobin levels from baseline to week 16, along with increased transfusion independence. BTH occurred in 10% of patients receiving pegcetacoplan compared to 23% in the eculizumab group. One case of sepsis was reported in the pegcetacoplan arm as a serious adverse event [47]. The most common adverse events included injection site reactions, diarrhea, headache, and fatigue. Importantly, no cases of meningitis or thromboembolic events were observed. Despite targeting a broader segment of the cascade—which could increase the risk of infections by encapsulated organisms—most reported infections in both treatment arms were mild upper respiratory tract infections or SARS-CoV-2 infections [47].

Subsequent studies have reinforced pegcetacoplan’s role as an effective therapeutic option in PNH. However, acute BTH remains a concern, possibly related to increased RBC survival and larger PNH clone sizes, which may alter the dynamics of complement activation [29,48].

The following Table 1 provides a comparative overview of key complement inhibitors currently used or in development for the treatment of PNH. It highlights their molecular targets, routes of administration, dosing intervals, main clinical advantages, and known limitations based on trial data and clinical experience. This summary supports phenotype-driven and individualized therapy selection.

#### 5.5.2. Factor B Inhibitors

Iptacopan is a highly selective, orally administered small-molecule inhibitor of factor B (FB), a key enzyme of the alternative complement pathway (AP). By targeting FB, iptacopan inhibits the formation of both the C3 convertase (C3Bb) and the C5 convertase (C3bC3bBb) on the surface of RBCs, thereby controlling both EVH and IVH driven by alternative pathway activation. Iptacopan has a relatively short plasma half-life of approximately 20 h and is administered orally at a dose of 200 mg twice daily. It has shown promising efficacy as monotherapy in both treatment-experienced and treatment-naïve patients.

The pivotal APPLY-PNH trial demonstrated that iptacopan was superior to C5 inhibitors in patients with inadequate response to eculizumab or ravulizumab, achieving sustained increases in hemoglobin and improved transfusion independence. Its safety profile was comparable to that of C5 inhibitors: most infections were mild to moderate, including upper respiratory and gastrointestinal tract infections, with no reported cases of meningococcal disease. Notably, only 2 out of 62 patients in the iptacopan arm experienced BTH [49]. Furthermore, the APPOINT-PNH trial confirmed the efficacy and tolerability of iptacopan in a treatment-naïve population, with the study meeting its primary endpoint of hemoglobin improvement and/or transfusion independence [50,51].

#### 5.5.3. Factor D Inhibitors

Factor D (FD) is a serine protease predominantly produced by adipocytes and plays a critical role in activating the AP. It also contributes secondarily to the CP and LP via the amplification loop. FD cleaves factor B (FB) when it is bound to C3(H_2_O) or C3b, generating the active C3 convertases C3(H_2_O) Bb and C3bBb. Inhibiting FD prevents the AP-mediated cleavage of C3 into C3a and C3b, thereby blocking the formation of downstream convertases and the terminal complement cascade [52,53]. Several agents targeting FD have been developed, including danicopan, vemircopan, and pelecoptan. Among these, danicopan is the most clinically advanced, and the only orally available FD inhibitor with robust data supporting its use in PNH treatment [54].

Danicopan acts by reversibly binding to FD, thereby preventing the formation of the AP C3 convertase. It has a relatively short half-life (6–8 h), and typical dosing in clinical trials has ranged from 50 mg to 200 mg three times daily. The FDA approved danicopan as an add-on therapy for patients receiving C5 inhibitors (eculizumab or ravulizumab) who remain anemic. This combination strategy, pairing terminal and proximal pathway blockade, is particularly effective in controlling both EVH and BTH.

The pivotal ALPHA trial—a Phase III, randomized, double-blind, placebo-controlled study—evaluated danicopan in patients with clinically significant EVH despite C5 inhibition. The study demonstrated significant improvements in hematologic parameters, including increased hemoglobin levels, reduced reticulocyte counts, lower LDH concentrations, decreased transfusion requirements [55,56]. Adverse events were generally manageable, and danicopan offers a convenient oral administration route, enhancing its appeal in clinical practice.

## 6. Future Directions

### 6.1. Novel Therapeutic Approaches

The therapeutic landscape for PNH is continuously evolving, with novel agents aiming to address unmet needs by improving the convenience of administration and enhancing patients’ quality of life [57].

### 6.2. Next Generation C5 Inhibitors

Emerging therapies are designed to optimize the efficacy of terminal complement inhibition, aiming to further reduce IVH, minimize BTH, and improve treatment convenience.

- Pozelimab (REGN3918): A fully human IgG4 monoclonal antibody targeting C5, offering potential advantages over first-generation agents. In the phase II trial (NCT03946748), pozelimab demonstrated rapid and sustained IVH control with a favorable safety profile in both treatment-naïve patients and those previously off C5 inhibitors. It is administered as a single IV loading dose (30 mg/kg) followed by weekly subcutaneous injections (800 mg). Importantly, it has shown efficacy in patients with C5 polymorphisms and is also under evaluation in combination regimens [58].
- Zilucoplan (RA101495): A small, synthetic macrocyclic peptide that binds C5 with high specificity, allowing daily subcutaneous self-administration. It inhibits MAC formation by preventing C5 cleavage. Two phase II trials (studies 201 and 203) assessed its efficacy in eculizumab-naïve and switch cohorts. While efficacy in naïve patients was encouraging, responses in patients previously treated with C5 inhibitors were comparatively less robust. Its favorable safety profile and pharmacokinetics support further investigation, possibly in combination with proximal inhibitors [59].
- Cemdisiran (ALN-CC5): An RNA interference (RNAi) therapeutic that silences C5 production at the mRNA level in hepatocytes, reducing circulating C5 protein. As monotherapy, it provides partial control of IVH. Ongoing trials are evaluating its combination with pozelimab and other agents for improved complement inhibition [56,60,61].
- Tesidolumab (LFG316): A human IgG1/λ monoclonal antibody binding to a distinct C5 epitope from eculizumab or ravulizumab. This may benefit patients with C5 variants or C5 inhibitor resistance. A phase II trial confirmed its efficacy in both wild-type and variant C5 populations, meeting the primary endpoint of LDH reduction [3,62].

### 6.3. Alternative Pathway Inhibitors

- Zaltenibart (OMS906): A fully humanized IgG4 antibody targeting MASP-3, the main activator of factor D in the alternative pathway. Zaltenibart may reduce infection risk while offering robust EVH and IVH control. It is under phase II and III investigation in both treatment-naïve and C5-experienced patients [63,64].
- Properdin inhibitors (NM3086 and NM5072): Properdin (factor P) stabilizes C3/C5 convertases in the AP. Inhibiting properdin interrupts C3 deposition on RBCs, offering a new strategy for EVH control. These agents are in early-phase clinical development (Phase I) [65,66].

### 6.4. Eculizumab Biosimilars

Biosimilars are biologic agents with comparable efficacy and safety to approved reference drugs. In PNH, two biosimilars of eculizumab have been authorized by the FDA: Bkemv (Eculizumab-aeeb) and Epysqli (eculizumab-aagh) [67]. Bkemv or ABP 959 was evaluated in the DAHLIA phase III, randomized double-blind, active-controlled trial, where it showed comparable efficacy, safety, and immunogenicity to eculizumab in patients with prior C5 inhibitor exposure [68]. Epysqli or SB12 demonstrated equivalent control of IVH (as measured by LDH levels) and safety in its pivotal phase III trial [69]. While promising, biosimilars require further real-world data to define their exact role in clinical practice.

### 6.5. Combination Therapies

Combining complement inhibitors with different targets may enhance efficacy and minimize residual hemolysis.

Pozelimab + Cemdisiran: This combination is under evaluation in multiple clinical trials. In the NCT05133531 phase III study, it was compared to ravulizumab in treatment-naïve patients. Interim analysis showed superior LDH reduction and IVH control versus the standard of care [70].

## 7. Unmet Needs in PNH

Despite significant therapeutic advances, critical challenges remain in the comprehensive management of PNH:

- Incomplete control of EVH: A subset of patients treated with C5 inhibitors continues to demonstrate residual anemia and transfusion dependency, despite effective blockade of IVH. Emerging proximal complement inhibitors may address this gap, but comparative data are still limited, and the long-term clinical benefit of fully controlling EVH remains to be established [8].
- Safety of proximal complement inhibitors: While proximal inhibitors provide broader control of hemolysis, they are associated with increased susceptibility to infections, particularly from encapsulated bacteria, underscoring the need for enhanced safety profiles, vaccination strategies, and appropriate antimicrobial prophylaxis. Long-term safety data remain limited, and ongoing surveillance is required to assess the risks of chronic complement pathway blockade in real-world practice [33].
- Access affordability and adherence: The high cost of complement inhibitors restricts equitable treatment access in many regions, particularly in resource-constrained settings. Even in high-income countries, cost-effectiveness and reimbursement policies vary widely, creating disparities in availability. Moreover, the requirement for lifelong therapy, intensive monitoring, and regular intravenous or subcutaneous administration further contributes to healthcare burden and may compromise long-term adherence. Strategies to reduce costs, simplify route and frequency of administration, and expand global access are urgently needed.
- Lack of validated biomarkers: Predictive markers for guiding treatment selection, predicting response, and monitoring disease activity are currently lacking, hindering the implementation of personalized therapy paradigms. Although potential biomarkers such as C3 fragment deposition on erythrocytes, plasma free hemoglobin, complement activity assays, and high-sensitivity flow cytometry parameters are under investigation, none have yet been standardized or validated for routine clinical use. The absence of reliable biomarkers delays individualized treatment decisions and complicates the development of prognostic models [71].
- Pregnancy: PNH during pregnancy remains a uniquely challenging scenario. Among current therapies, eculizumab is the only extensively studied and routinely used option, with evidence supporting improved maternal and fetal outcomes. However, breakthrough hemolysis, variable pharmacokinetics during pregnancy, and limited data on optimal dosing remain concerns. Ravulizumab may represent a safe and effective option for pregnant women with PNH, but larger prospective studies and systematic data collection are needed to confirm safety and guide clinical practice. Evidence regarding the use of proximal complement inhibitors remains preliminary and insufficient [72].
- Pediatric PNH: In children and young adults, disease characteristics and treatment responses remain poorly characterized, with therapeutic decisions extrapolated from adult experience, underscoring the need for dedicated clinical trials in these vulnerable populations. Evidence from registries indicates that eculizumab and ravulizumab are effective with an acceptable safety profile in children. Additional challenges include the impact on growth and development, fertility considerations, and the role of hematopoietic stem cell transplantation in select cases. Ongoing research and dedicated pediatric registries are critical to optimizing care and tailoring therapeutic strategies for this subgroup [73].
- Prognostic models: The absence of a validated prognostic model to stratify patients according to risk of relapse or treatment failure represents a major gap. A robust prognostic tool could integrate multiple clinically relevant parameters including the size of the PNH clone, markers of hemolysis (e.g., LDH, hemoglobin, reticulocyte count), evidence of C3 fragment deposition, bone marrow status, history of thrombosis. Stratifying patients into low, intermediate, and high-risk categories could inform treatment escalation strategies, identify candidates for HSCT or proximal inhibitors, and guide intensified monitoring. The integration of NGS into risk stratification models remains also an unmet need. Somatic mutations beyond *PIGA*, such as those in BCOR, BCORL1, DNMT3A, or ASXL1, may influence clonal dynamics, prognosis, and response to therapy, but their clinical utility is not yet standardized. Incorporating NGS data into validated prognostic tools could refine risk assessment and support more individualized treatment strategies. However, the low global prevalence of PNH poses a major barrier to developing and validating such algorithms through large, prospective datasets. However, the low global prevalence of PNH poses a major barrier to developing and validating such algorithms through large, prospective datasets.

## 8. Discussion

The management of PNH illustrates the complex interplay of the complement cascade in disease pathogenesis and therapy. The advent of complement inhibitors has transformed PNH from a life-threatening disorder into a chronic, manageable condition. Before the introduction of C5 inhibitors (C5i), median survival was limited to 10–15 years after diagnosis, with thrombosis being the leading cause of early mortality, while treatment options were limited to supportive care, transfusions, and, in selected cases, allogeneic HSCT, typically reserved for younger, fit patients.

Over the past two decades, the therapeutic landscape has undergone a paradigm shift, first with eculizumab, then with ravulizumab, both of which target terminal complement activation. These agents have markedly improved overall survival by effectively controlling IVH, leading to reduced transfusion requirements, fewer thrombotic events, improved fatigue, and enhanced quality of life. Nevertheless, limitations persist, such as BTH, persistent EVH, the need for lifelong intravenous infusions, and substantial treatment costs.

While C5 inhibitors remain foundational, emerging agents and combination strategies are expanding therapeutic possibilities. For instance, crovalimab, a next-generation C5i, offers subcutaneous administration and extended dosing intervals without compromising efficacy or safety. Additionally, biosimilars of eculizumab (e.g., Bkemv, Epysqli) have shown comparable efficacy, pharmacokinetics, and safety profiles, potentially increasing accessibility and cost-effectiveness.

Moreover, proximal complement inhibitors, including C3 inhibitors (pegcetacoplan), FD inhibitors (danicopan), and FB inhibitors (iptacopan), provide broader control of complement activation, addressing both IVH and EVH. However, while these agents show promising hemoglobin stabilization and transfusion independence in clinical trials, the generalizability of these results remains uncertain due to small sample sizes, exclusion of patients with severe marrow failure, and limited long-term safety data. For example, the PEGASUS and APPLY-PNH trials, although pivotal, included selected populations under controlled conditions, and their applicability to real-world, comorbid patients requires further investigation. Moreover, the broader inhibition of upstream complement components may increase infection risk, warranting caution in immunocompromised patients and necessitating strict vaccination protocols. Preliminary data suggest these agents can reduce transfusion needs, improve hemoglobin levels, and enhance patient-reported outcomes. Combination regimens, targeting multiple complement components, are emerging as a promising strategy for comprehensive blockade and resistance prevention.

As we move toward precision medicine, research will increasingly focus on refining these therapies for maximal efficacy with minimal toxicity. Key priorities include optimizing dosing regimens, reducing administration burden, improving global access to therapy, minimizing adverse effects.

## 9. Conclusions

In conclusion, PNH has evolved from a life-threatening hematologic disorder into a chronic, manageable disease with substantially improved survival in the era of complement inhibition. Emerging therapies are driving the field toward more personalized, convenient, and accessible care, with innovative agents poised to further redefine therapeutic goals and address the remaining limitations of current treatments.

Real-world data from international registries are critical to shaping clinical practice and capturing long-term outcomes. Additionally, developing prognostic models to identify patients at risk for loss of therapeutic response, using markers such as PNH clone size, LDH, reticulocyte count, and history of thrombosis, or even mutational status, could guide individualized treatment decisions. However, the rarity of PNH remains a major barrier, underscoring the importance of multinational collaboration and the integration of large datasets.

## Figures and Tables

**Table 1:** Summary of Complement Inhibitors in PNH. IV: intravenous, SC: subcutaneous, IVH: intravascular hemolysis, BTH: breakthrough hemolysis, EVH: extravascular hemolysis, Hb: hemoglobin, Ab: antibody, BID: twice daily, TID: three times daily.

Agent	Target	Route	Dosing Interval	Key Benefits	Limitations
Eculizumab	C5	IV	Biweekly	Reduces IVH, improves survival	BTH and EVH, IV only
Ravulizumab	C5	IV	Every 8 weeks	Longer half-life, fewer BTH events	Cost, infection risk, residual EVH, IV only
Crovalimab	C5 (recycling Ab)	SC	Monthly	SC, convenient, less frequent dosing, robust IVH control	Limited long-term data, infection risk
Pegcetacoplan	C3	SC	Twice weekly	SC, controls both IVH and EVH	Injection site reactions, infection risk, “massive hemolysis”
Iptacopan	Factor B	Oral	BID	Improves Hb, oral route, monotherapy	Mild infections, long-term safety under review
Danicopan	Factor D	Oral	TID	Add-on therapy, oral route, effective in EVH	Short half-life, TID dosing

## Data Availability

Not applicable.
